# Caregiver Identity and Resilience Across and Beyond Dementia Care: A Qualitative Study

**DOI:** 10.1093/geroni/igaf122.3892

**Published:** 2025-12-31

**Authors:** Ida Ghaemmaghamfarahani, Chizobam Nweke, R Amanda Cooper

**Affiliations:** University of Connecticut, Storrs, Connecticut, United States; University of Connecticut, Storrs, Connecticut, United States; University of Connecticut, Storrs, Connecticut, United States

## Abstract

With increasing dementia prevalence and reliance on spousal care partners, understanding how caregiving identity evolves and how resilience is fostered across the care trajectory is crucial. While dementia research often emphasizes caregiver burden, fewer studies explore how identity and resilience develop in response to shifting roles and changing relationship dynamics. Guided by Caregiver Identity Theory, we conducted a thematic analysis of 18 in-depth interviews with long-term spousal caregivers of people living with dementia to examine how they experience identity transitions and cultivate resilience as care dynamics evolve. Five interrelated themes emerged: disruption of familiar roles and relational structures, highlighting loss of shared routines and partner reciprocity; internalizing the caregiver identity, reflecting deepened commitment and sense of duty; navigating uncertainty and strain, encompassing emotional, physical, and existential stressors, including ambiguous loss and caregiving self-doubt; sustaining identity through adaptation and engagement, illustrating how caregivers reframed challenges, sought support, and retained purpose; and meaning-making through connection and shared history, capturing how long-term bonds, lucid moments, and partner appreciation contributed to resilience and meaning. Findings highlight caregiving identity as a dynamic, evolving process. Caregivers developed resilience by adapting to changing roles, remaining grounded in personal values, and making meaning of their experiences over time. For some, caregiver identity continued beyond active care, offering insight into how long-term roles and meaning-making evolve. These insights can inform research and guide efforts to better understand and support caregivers’ evolving identities, needs, and processes through which they construct resilience and meaning across and beyond the care journey.

